# Prognostic value of complement serum C3 level and glomerular C3 deposits in anti-glomerular basement membrane disease

**DOI:** 10.3389/fimmu.2023.1190394

**Published:** 2023-07-05

**Authors:** Pauline Caillard, Cécile Vigneau, Jean-Michel Halimi, Marc Hazzan, Eric Thervet, Morgane Heitz, Laurent Juillard, Vincent Audard, Marion Rabant, Alexandre Hertig, Jean-François Subra, Vincent Vuiblet, Dominique Guerrot, Mathilde Tamain, Marie Essig, Thierry Lobbedez, Thomas Quemeneur, Mathieu Legendre, Alexandre Ganea, Marie-Noëlle Peraldi, François Vrtovsnik, Maïté Daroux, Raïfah Makdassi, Gabriel Choukroun, Dimitri Titeca-Beauport

**Affiliations:** ^1^Department of Nephrology, Dialysis, and Transplantation, University of Picardie Jules Verne, Amiens University Hospital, Amiens, France; ^2^Mécanismes Physiopathologiques et Conséquences des Calcifications Cardiovasculaires (MP3CV) laboratory, Centre de Recherche en Santé (CURS), Amiens, France; ^3^Rennes University Hospital, Inserm, Ecole des hautes études en santé publique (EHESP), Irset (Institut de recherche en santé, environnement et travail) - UMR_S 1085, Rennes, France; ^4^Department of Nephrology, Tours University Hospital and EA4245, University of Tours, Tours, France; ^5^Nephrology Department, Lille University Hospital, University of Lille, UMR 995, Lille, France; ^6^Department of Nephrology, Georges Pompidou European Hospital, Assistance Publique-Hôpitaux de Paris (APHP), Paris and INSERM UMRS970, Boulogne-Billancourt, France; ^7^Department of Nephrology and Dialysis, Annecy Genevois Hospital, Pringy, France; ^8^Department of Nephrology, Edouard Herriot Hospital, Hospices Civils de Lyon, Carmen INSERM 1060 and Univ Lyon, Lyon, France; ^9^Department of Nephrology and Renal Transplantation, Reference Center-Idiopathic Nephrotic Syndrome, Henri-Mondor Hospital/Albert-Chenevier, Assistance Publique-Hôpitaux de Paris (AP-HP) Créteil, INSERMU955, Paris Est Créteil University, Créteil, France; ^10^Pathology Department, Necker University Hospital, Assistance Publique-Hôpitaux de Paris (AP-HP). Centre-Université de Paris, Paris, France; ^11^Department of Nephrology, Dialysis and Transplantation, Foch Hospital, Paris-Saclay University, Suresnes, France; ^12^Department of Nephrology, Dialysis and Transplantation, University Hospital, Angers and Centre de Recherche en Cancérologie et Immunologie Nantes-Angers (CRCINA), INSERM, Nantes University, Angers University, Angers, France; ^13^Department of Nephrology and Renal Transplantation, Reims University Hospital, Reims, France; ^14^Department of Nephrology, Rouen University Hospital, Rouen and INSERM, U1096 Rouen, France; ^15^Department of Nephrology and Dialysis, Vichy Hospital, Vichy, France; ^16^Department of Nephrology, Dialysis, and Renal Transplantation, Ambroise-Paré Hospital, Assistance Publique-Hôpitaux de Paris (AP-HP), Paris-Saclay University, Boulogne-Billancourt, France; ^17^Department of Nephrology, Caen University Hospital, Caen, France and the French Registry of Peritoneal Dialysis, Langue Française, Pontoise, France; ^18^Department of Nephrology and Internal Medicine, Valenciennes General Hospital, Valenciennes, France; ^19^Department of Nephrology, Dialysis and Renal Transplantation, University Hospital, Dijon, France; ^20^Department of Nephrology, Orleans Hospital, Orleans, France; ^21^Department of Nephrology, Dialysis and Renal Transplantation, Necker University Hospital, Assistance Publique-Hôpitaux de Paris (AP-HP), Centre-Université de Paris, Paris, France; ^22^Nephrology Department, Bichat-Claude Bernard Hospital, APHP, Paris, France. Faculty of Medicine, Paris Diderot University, Sorbonne Paris Cité, Paris, France; ^23^Department of Nephrology, Duchenne Hospital, Boulogne-Sur-Mer, France

**Keywords:** anti-glomerular basement membrane disease, complement C3, C3 glomerular deposits, kidney biopsy, kidney prognosis, kidney survival

## Abstract

**Background and objectives:**

Activation of the complement system is involved in the pathogenesis of anti-glomerular basement membrane (anti-GBM) disease. Glomerular deposits of complement 3 (C3) are often detected on kidney biopsies. The primary objective of this study was to analyze the prognostic value of the serum C3 level and the presence of C3 glomerular deposits in patients with anti-GBM disease.

**Methods:**

We conducted a retrospective cohort study of 150 single-positive patients with anti-GBM disease diagnosed between 1997 and 2017. Patients were categorized according to the serum C3 level (forming a low C3 (C3<1.23 g/L) and a high C3 (C3≥1.23 g/L) groups) and positivity for C3 glomerular staining (forming the C3+ and C3- groups). The main outcomes were kidney survival and patient survival.

**Results:**

Of the 150 patients included, 89 (65%) were men. The median [interquartile range (IQR)] age was 45 [26–64]. At diagnosis, kidney involvement was characterized by a median [IQR] peak serum creatinine (SCr) level of 578 [298–977] µmol/L, and 106 (71%) patients required dialysis. Patients in the low C3 group (72 patients) had more severe kidney disease at presentation, as characterized by higher prevalences of oligoanuria, peak SCr ≥500 µmol/L (69%, vs. 53% in the high C3 group; p=0.03), nephrotic syndrome (42%, vs. 24%, respectively; p=0.02) and fibrous forms on the kidney biopsy (21%, vs. 8%, respectively; p=0.04). Similarly, we observed a negative association between the presence of C3 glomerular deposits (in 52 (41%) patients) and the prevalence of cellular forms (83%, vs. 58% in the C3- group; p=0.003) and acute tubulo-interstitial lesions (60%, vs. 36% in the C3- group; p=0.007). When considering patients not on dialysis at diagnosis, the kidney survival rate at 12 months was poorer in the C3+ group (50% [25-76], vs. 91% [78-100] in the C3- group; p=0.01), with a hazard ratio [95% confidence interval] of 5.71 [1.13-28.85] (p=0.04, after adjusting for SCr).

**Conclusion:**

In patients with anti-GBM disease, a low serum C3 level and the presence of C3 glomerular deposits were associated with more severe disease and histological kidney involvement at diagnosis. In patients not on dialysis at diagnosis, the presence of C3 deposits was associated with worse kidney survival.

## Introduction

Anti-glomerular basement membrane (anti-GBM) disease is a type of small-vessel vasculitis mediated by autoantibodies against the non-collagenous domain of the α3 chain of type IV collagen (1, 2). Depending on various factors (age, exposure to toxic substances, etc.), both the kidneys and lungs can be damaged, with rapidly progressing glomerulonephritis and/or diffuse alveolar hemorrhage (3, 4). The rapid initiation of plasma exchange with corticosteroids (CSTs) and cyclophosphamide is associated with longer kidney survival and overall survival (5, 6). Despite the provision of standardized treatment, kidney survival is still very poor in patients presenting with a serum creatinine (Scr) level ≥ 500 µmol/l, anuria, dependence on dialysis, or a high percentage of crescents in the kidney biopsy (7–9). Preclinical and clinical studies have highlighted the role of both classical and alternative complement pathways in the pathogenesis of anti-GBM disease (10–14). Recently, an association between the serum C3 level, C3 glomerular deposits, and poor kidney survival was observed in two retrospective, single-center studies (15, 16). The complement pathway is an important component of the innate and adaptive immune systems (17) and is involved in glomerular diseases such as systemic lupus nephritis, antineutrophil cytoplasmic antibody (ANCA)-associated vasculitis (AAV), C3 glomerulopathy, and atypical hemolytic uremic syndrome (18–23). In AAV, a low serum C3 level at diagnosis is associated with more severe kidney involvement and worse kidney and overall survival rates (24–26). C3 deposition in the kidney is also linked to more severe renal injury at diagnosis (25, 27). The role of other complement fractions has been also highlighted in the literature (28, 29). The putative influence of the complement pathway has also been investigated in immunoglobulin A nephropathy (IgAN) and membranous nephropathy; in IgAN, a low serum C3 was associated with a poor kidney outcome (30, 31). There is growing interest in the development of treatments that target the complement pathway in AAV and other glomerulopathies; examples include the recent development of a complement 5a receptor antagonist (avacopan) in AAV, an anti-factor B agent (iptacopan) in C3 glomerulopathy, and an anti-C5 agent (ravulizumab) and an anti-C3 agent (pegcetacoplan) in lupus nephritis) (32–35). However, the clinical consequences of complement activation in anti-GBM disease have not been extensively investigated.

The primary objective of the present study was to determine the prognostic value of the serum C3 level and the presence of C3 glomerular deposits in adults with anti-GBM disease.

## Materials and methods

### The study cohort

We retrospectively analyzed data from 205 patients diagnosed with anti-GBM disease in the nephrology departments of 22 French centers (19 university medical centers and 3 tertiary hospitals) between January 1997 and December 2017. We screened all patients presenting with clinical manifestations of rapidly progressive glomerulonephritis and with glomerular linear immunoglobulin (Ig) deposits on renal biopsy and/or positive serum anti-GBM antibodies. Double-positive patients presenting anti-GBM disease (glomerular linear Ig deposits) and a positive ANCA assay (using an indirect immunofluorescence assay and/or an antigen-specific immunoassay) were excluded from the study. The time of diagnosis was defined as the date on which anti-GBM disease was first detected using a serological test or a histological assessment. The study protocol was approved by the local institutional review board (*CPP Nord Ouest II*, Amiens, France; reference: TB/LR/2016–91).

### Clinical data

Patients were considered to have entered the study on the date when anti-GBM disease had been diagnosed. All the patients were diagnosed at hospital (on conventional wards or in intensive care units). The time interval between hospital admission and diagnosis (using a serological test or a histological assessment) was noted. Clinical and laboratory data at presentation and during follow-up were retrieved from each center’s medical records. Serum levels of C-reactive protein and albumin were measured using a standard liquid-phase immunoassay. Complement fractions (C3 and C4) were measured using an immunoturbidimetric ELISA. All the light microscopy findings (the percentage of crescents and the presence of tubular and interstitial damage) and immunofluorescence findings (the type and location of Ig and/or complement deposits) were noted. The kidney biopsies were also rated according to Berden et al.’s classification (focal, cellular, fibrous, or mixed) and Brix et al.’s risk score (depending on the percentage of normal glomeruli and tubulo-interstitial lesions) (36, 37). The cohort was divided into groups with regard to the median serum C3 level of 1.23 g/L [thus forming a low C3 group (serum C3<1.23 g/L) and a high C3 group (serum C3 ≥1.23 g/L)] and the presence or absence of linear C3 deposits on the GBM in an immunofluorescence analysis (thus forming the C3+ and C3- groups, respectively). Diffuse alveolar hemorrhage was defined as the presence of diffuse, bilateral, parenchymal infiltrates on chest imaging, together with hemoptysis or the visual detection of bleeding during a bronchoalveolar lavage (38). Hypoxemic respiratory failure was defined as the requirement for an oxygen flow rate ≥6 L/min for the maintenance of a blood oxygen saturation level ≥92%. Intensive treatment was defined as the combination of CSTs, plasma exchange (PLEX), and immunosuppressive agents (IMSs: cyclophosphamide and/or rituximab) (CSTs+PLEX+IMS). Dialysis dependency at presentation was defined as the need for renal replacement therapy (RRT) during the initial hospital stay. End-stage kidney disease (ESKD) was defined as RRT for at least 12 weeks until last follow up or kidney transplantation. In patients who required dialysis at presentation, renal recovery was defined as being weaned off RRT during for at least 12 weeks during the follow-up period. The patient survival time was defined as the time interval between diagnosis and death. The date of last follow-up corresponded to the date of the patient’s death or the last visit before the end of the study (December 31^st^, 2017). The primary endpoint was the kidney survival rate (i.e. the absence of ESKD) at 12 months, and the secondary endpoint was the patient survival rate at 36 months.

### Statistical analyses

The patients’ characteristics were summarized as the frequency (%) for categorical variables and the median [interquartile range (IQR)] for continuous variables. In comparisons of the low vs. high serum C3 groups and the C3+ vs. C3- deposit groups, categorical variables were compared in a chi-squared test and quantitative variables were compared in a Mann-Whitney U test. Survival was assessed using the Kaplan-Meier method. To assess the factors associated with the progression to ESKD, univariate and multivariate Cox proportional hazards regression analyses were performed. The threshold for statistical significance was set to *p* < 0.05. All statistical analyses were performed with MedCalc® software (version 19.0.6, MedCalc Software Ltd, Ostend, Belgium).

## Results

### Baseline characteristics of the study participants

**Figure 1:**
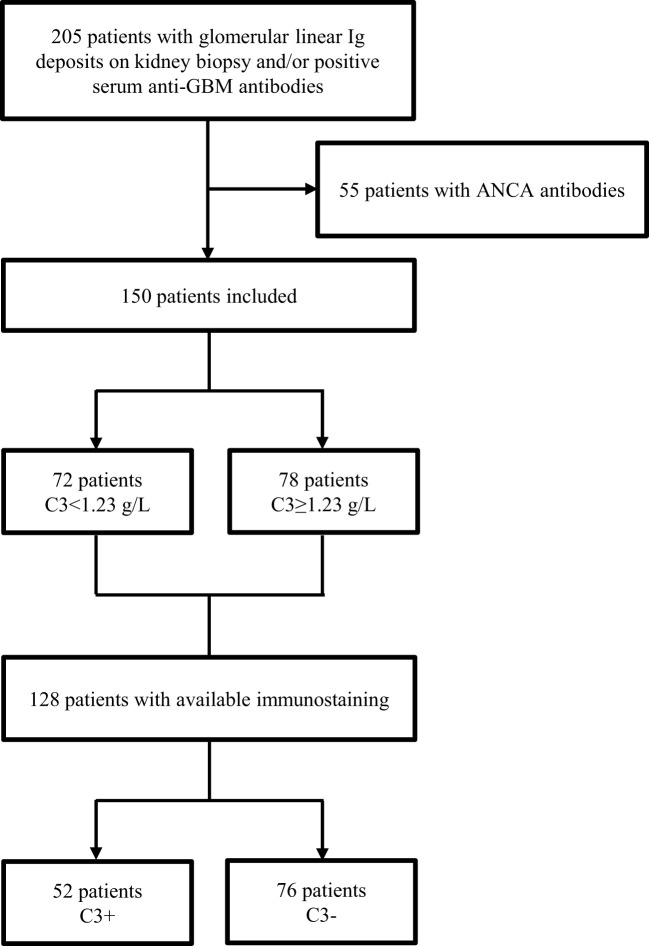
Study flow chart.

Of the 205 patients diagnosed with anti-GBM disease, 150 were included in the study (**Figure 1**). All the 150 patients were diagnosed at hospital, including 20 (13%) in an intensive care unit. The median [IQR] time interval between hospital admission and diagnosis was 5 [4-9] days.

The baseline demographic and clinical characteristics of the study participants are summarized as a function of the serum C3 level in **Table 1**. The cohort comprised 89 men (65%), and the median [IQR] age at diagnosis was 45 [26–64]. Tobacco use (49%) and chronic arterial hypertension (33%) were the most frequent comorbidities. The median [IQR] peak SCr level was 578 [298–977] µmol/L, 91 (61%) patients presented oligoanuria, and 106 (71%) patients required dialysis at presentation. A total of 71 patients (47%) had a proteinuria value >3 g/24 h, and 49 (33%) presented with nephrotic syndrome. Fifty-nine (39%) patients had diffuse alveolar hemorrhage, and 30 of these (51%) also had acute respiratory distress. The median [IQR] serum C3 level was 1.23 [1.10-1.37] g/L: hence, 72 patients had a low C3 level, and 78 had a high C3 level.

**Table 1:** Characteristics of the study participants at diagnosis.

	Total (n=150)	C3 <1.23 g/L (n=72)	C3 ≥1.23 g/L (n=78)	*p*-value
Age	45 [26–64]	40 [23-61]	51 [27–64]	0.26
Males	89 (65)	43 (60)	46 (59)	0.93
Comorbidities				
Diabetes mellitus	9 (6)	6 (8)	3 (4)	0.25
Chronic arterial hypertension	49 (33)	26 (36)	23 (29)	0.39
Dyslipidemia	28 (19)	17 (24)	11 (14)	0.14
Tobacco use	74 (49)	34 (47)	40 (51)	0.62
Cardiovascular diseases	11 (7)	7 (10)	4 (5)	0.28
Cancer	14 (9)	9 (13)	5 (6)	0.20
Charlson Comorbidity Index	1 [0–3]	1 [0–3]	0 [0–1]	0.70
Performance status	1 [1–2]	1 [1–2]	1 [1–2]	0.47
Antibodies				
Anti-GBM	133 (89)	64 (89)	69 (88)	0.93
Antinuclear antibodies	32 (21)	19 (26)	13 (17)	0.15
Kidney involvement				
Oligoanuria	91 (61)	50 (69)	41 (53)	0.03
Arterial hypertension	89 (59)	45 (63)	44 (56)	0.45
Peak SCr (µmol/L)	578 [298–977]	680 [343-1055]	521 [276–870]	0.06
Peak SCr ≥500 µmol/L	91 (61)	50 (69)	41 (53)	0.03
Proteinuria >3.0 g/24 h	71 (47)	41 (57)	30 (38)	0.02
Nephrotic syndrome	49 (33)	30 (42)	19 (24)	0.02
Required dialysis at diagnosis	106 (71)	56 (78)	50 (64)	0.06
Lung involvement				
Alveolar hemorrhage	59 (39)	30 (42)	29 (37)	0.57
Acute respiratory distress	30 (51)	14 (47)	16 (55)	0.51
Mechanical ventilation	14 (47)	7 (50)	7 (44)	0.73
Laboratory parameters				
Serum albumin (g/L)	26.4 [22.6–30.0]	26.0 [22.0–29.1]	27.0 [23.0–30.3]	0.54
Leukocyte count (cells/mm^3^)	10.7 [8.5–13.3]	10.7 [8.4–14.4]	11 [8.7–12.5]	0.52
Hemoglobin (g/dL)	8.4 [7.4–9.7]	8.0 [7.2–9.5]	8.7 [7.5–9.8]	0.10
C-reactive protein (mg/L)	102 [25–179]	65 [20–181]	118 [50–178]	0.09
C3 (g/L)	1.23 [1.10-1.37]	1.09 [0.95-1.15]	1.37 [1.27-1.53]	<0.0001
C4 (g/L)	0.29 [0.23-0.35]	0.25 [0.20-0.29]	0.32 [0.27-0.39]	<0.0001

Values are expressed as the median [interquartile range] or the number (percentage). Anti-GBM, antibodies against glomerular basement membrane; SCr, serum creatinine.

A total of 135 patients underwent a kidney biopsy, and immunostaining data were available for 128 (95%) of these. The cellular form was predominant (68%), 63 (49%) patients had fewer than 10% of normal glomeruli, and 58 (45%) patients had acute tubulo-interstitial lesions (**Table 2**). All 128 biopsies had Ig linear GBM deposits: 115 (91%) with IgG alone, one (0.8%) with IgA alone, and 12 (9%) with two of more types of Ig (i.e. IgG/IgA/IgM). Linear C3 GBM deposits were observed in 52 (41%) patients, and C1q GBM deposits were observed in 4 (3%) patients.

**Table 2:** Kidney biopsy findings at diagnosis, as a function of the serum C3 level.

	Total (n=128)	C3 <1.23 g/L (n=63)	C3 ≥1.23 g/L (n=65)	*p*-value	
**Immunofluorescence**	128 (100)	63 (100)	65 (100)		
IgG linear GBM deposits	127 (99)	62 (98)	65 (100)	0.31	
IgM linear GBM deposits	7 (6)	3 (5)	4 (6)	0.73	
IgA linear GBM deposits	7 (6)	5 (8)	2 (3)	0.23	
C3 linear GBM deposits	52 (41)	30 (48)	22 (34)	0.11	
C1q linear GBM deposits	4 (3)	3 (5)	1 (2)	0.37	
Light chain K/L GBM deposits	21 (16)	15 (24)	6 (9)	0.03	
Light microscopy					
Number of normal glomeruli* (%)	0 [0-17]	0 [0-11]	0 [0-23]	0.36	
<10% of normal glomeruli	63 (49)	31 (49)	32 (49)	1	
Classification					
-focal	15 (12)	6 (10)	9 (14)	0.45	
-cellular	87 (68)	39 (62)	48 (74)	0.15	
-fibrous	18 (14)	13 (21)	5 (8)	0.04	
-mixed	8 (6)	5 (8)	3 (5)	0.44	
Tubular necrosis and/or interstitial infiltrate	58 (45)	27 (43)	31 (48)	0.58	
Tubular atrophy and/or interstitial fibrosis	25 (20)	13 (21)	12 (19)	0.76	

Values are expressed as the median [interquartile range] or the number (percentage). GBM, glomerular basement membrane; K/L, kappa/lambda light chains. * 124 patients with available data: 60 in the low C3 level group and 64 in the high C3 level group.

### Patient management and disease outcomes

A total of 144 (96%) patients were treated actively, and 108 (75%) of these were treated intensively (**Table 3**). Almost all the patients received methylprednisolone pulse therapy, and 90 (67%) of the patients alive at 6 months were still on CSTs at that time. The median [IQR] number of plasma exchange sessions was 12 [9–15], and cyclophosphamide was the most frequently administered IMS (83%, vs. 6% for rituximab).

**Table 3:** Treatments.

	Total (n=150)	C3 <1.23 g/L (n=72)	C3 ≥1.23 g/L (n=78)	*p*-value	
Treated *	144 (96)	68 (94)	76 (97)	0.35	
Intensive treatment	108 (75)	47 (69)	61 (80)	0.08	
Methylprednisolone pulses	143 (99)	67 (99)	76 (100)	0.29	
	3 [3-3]	3 [3-3]	3 [3-3]	0.12	
CSTs at 3 months^$^	121 (89)	55 (87)	66 (90)	0.56	
CSTs at 6 months^$^	90 (67)	37 (60)	53 (74)	0.08	
Daily dose of CSTs at 6 months (mg)^#^	10 [10–15]	10 [10–20]	10 [9–15]	0.27	
CSTs at 12 months^$^	33 (25)	15 (25)	18 (26)	0.93	
Plasma exchange	116 (81)	50 (74)	66 (87)	0.04	
Number of plasma exchange sessions	12 [9–15]	12 [9–15]	12 [8–15]	0.71	
Cyclophosphamide	120 (83)	56 (82)	64 (84)	0.77	
Cumulative 6-month dose (mg/kg)	63 [31–101]	55 [29–89]	75 [35–114]	0.84	
Rituximab	9 (6)	3 (4)	6 (8)	0.39	
Co-trimoxazole prophylaxis	99 (69)	44 (61)	55 (71)	0.91	
**At least one serious infection in the first 12 months of follow-up**	79 (53)	38 (53)	41 (53)	0.98	

Values are expressed as the median [interquartile range] or the number (percentage). CST, corticosteroid. *Data for the 144 actively treated patients. ^#^90 patients on CSTs at 6 months. ^$^ reported as a percentage of the patients alive (n=136, with 63 in the low C3 group and 73 in the high C3 group at 3 months; n=134, with 62 in the low C3 group and 72 in the high C3 group at 6 months, and n=130 with 60 in the low C3 group and 70 in the high C3 group at 12 months).

The median [IQR] follow-up time was 59.9 [25.7-124.1] months. At 12 months, the kidney survival rate [95% confidence interval (CI)] was 29% [22-37]. The kidney prognosis was also poor in the subgroup of patients receiving intensive treatment, with a kidney survival rate [95%CI] of 30% [21-39] at 12 months. Among the patients on dialysis at diagnosis, 8 (8%) had been weaned off after a median [IQR] of 2.3 [1.0-5.0] months. In all, 32 (21%) patients died during the follow-up period, including 19 (59%) within 3 years of diagnosis. The main causes of death during the first 3 years were cardiovascular and infectious diseases (accounting for 37% and 32% of the cases, respectively).

### Comparison of the low C3 and high C3 groups

Patients in the low C3 group had more severe kidney involvement at diagnosis, as evidenced by higher prevalences of oligoanuria, a peak SCr level ≥500 µmol/L (69%, vs. 53% in the high C3 group; p=0.03), proteinuria (57%, vs. 38%, respectively; p=0.02), and nephrotic syndrome (42%, vs. 24%, respectively; p=0.02) (**Table 1**). A low C3 serum level was also associated with a lower serum C4 level (0.25 [0.20-0.29] g/L in the low C3 group, vs. 0.32 [0.27-0.39] g/L in the high C3 group; p<0.0001) and a higher prevalence of fibrous forms in the kidney biopsy (21%, vs. 8%, respectively; p=0.04) (**Table 2**). There was no difference in the presence of C3 linear deposits between the two groups.

**Figure 2:**
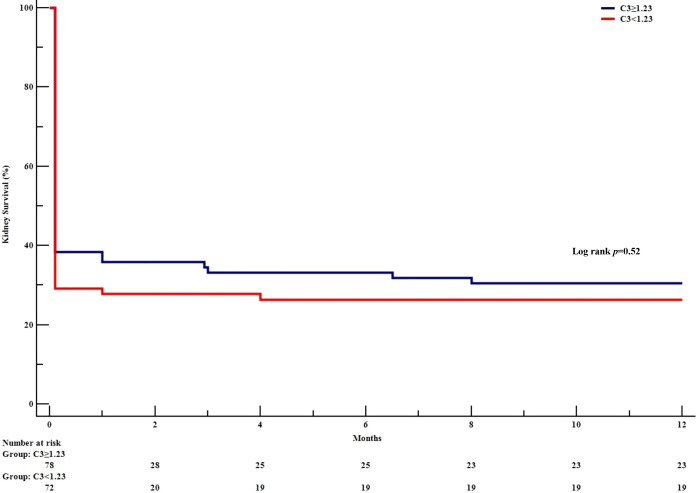
Kidney survival, as a function of the serum C3 level. The Kaplan-Meier survival curve shows the development of end-stage kidney disease in patients with anti-GBM disease. as a function of the serum C3 level.

Forty-seven (69%) of the patients in the low C3 group and 61 (80%) in the high C3 group (p=0.08) were treated intensively (**Table 3**). The two groups were treated in a similar manner, except that the proportion with plasma exchange was 74% in the low C3 group vs. 87% in the high C3 group (p=0.04). The kidney survival rates [95%CI] at 12 months were similar in the low and high C3 groups (26% [18-36] vs. 31% [22-42], respectively; p=0.52; **Figure 2**). During the first 3 years, 10 (14%) patients in the low C3 group and 9 (12%) patients in the high C3 group died; most of these deaths had cardiovascular and infectious causes.

### Comparison of patients with vs. without C3 kidney deposits

**Figure 3:**
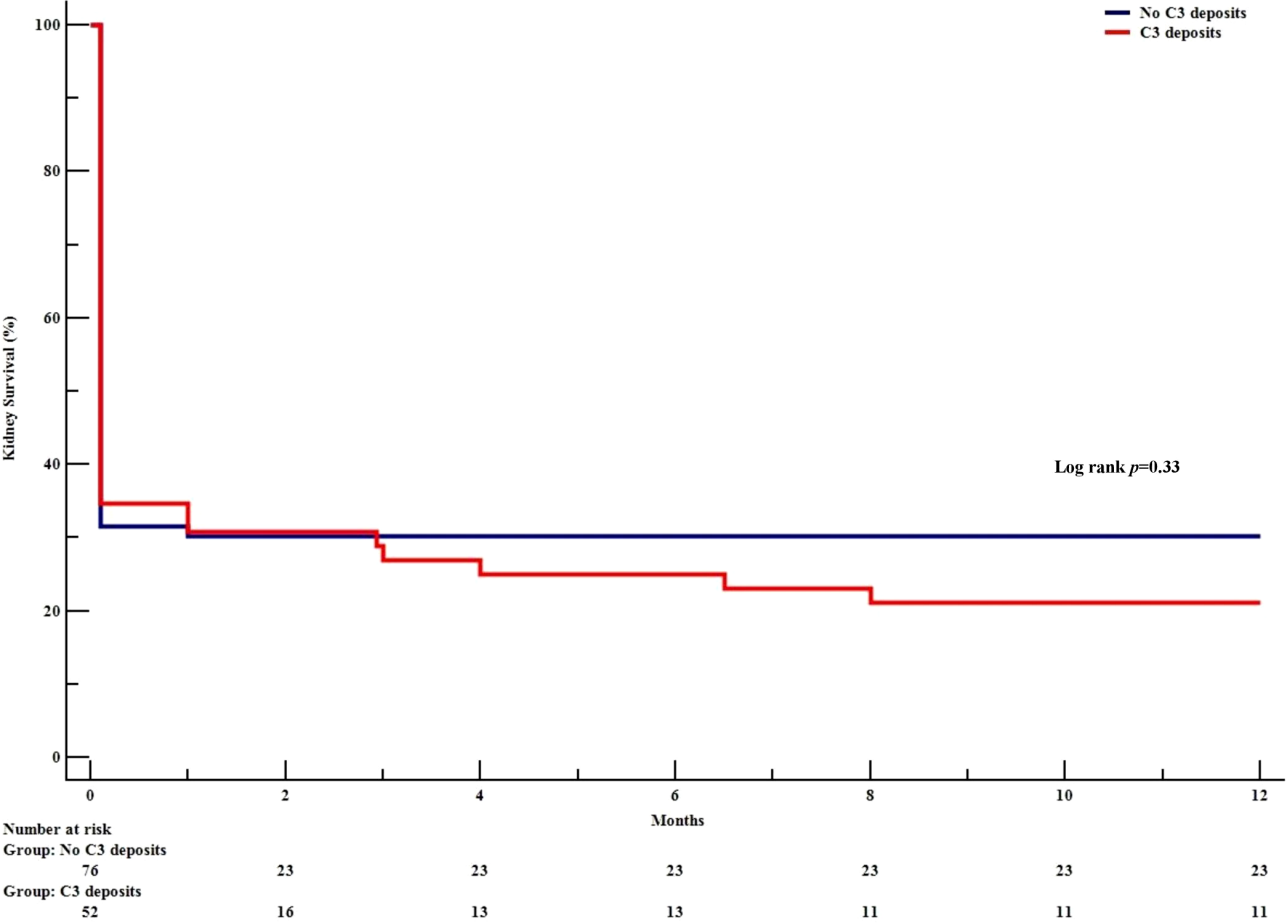
Kidney survival, as a function of the presence or absence of C3 GBM deposits. The Kaplan-Meier survival curve shows the development of end-stage kidney disease in patients with anti-GBM disease. as a function of the presence or absence of C3 GBM deposits.

Of the 128 patients with kidney biopsy immunostaining data, 52 (41%) presented C3 linear GBM deposits (forming the C3+ group; **Table 4**). 67% of the patients in the C3+ group had oligoanuria at baseline (vs. 59% in the C3- group), and the median [IQR] peak SCr level was 586 [385-1055] µmol/L (vs. 626 [313–975] µmol/L in the C3- group). Nephrotic-range proteinuria was present in 56% and 36% of the C3+ and C3- patients, respectively (p=0.02). The patients in the C3+ group had more severe histologic kidney lesions, as characterized by greater proportions of cellular forms (83%, vs. 58% in the C3- group; p=0.003) and acute tubulo-interstitial lesions (60%, vs. 36% in the C3- group; p=0.007). The median [IQR] serum C3 level was 1.18 [1.05-1.32] g/L in the C3+ group and 1.25 [1.10-1.42] g/L in the C3- group (p=0.06). The two groups did not differ with regard to their treatment. At 12 months, the kidney survival rate [95%CI] was 21% [10-32] in the C3+ group and 30% [20-40] in the C3- group (p=0.33) (**Figure 3**).

**Table 4:** Clinical and histological characteristics at diagnosis, as a function of the presence or absence of C3 kidney deposits.

			Total (n=128)		C3+ (n=52)		C3- (n=76)	*p*-value		
Age			47 [27–63]		45 [26-67]		49 [28–61]	0.84		
Antibodies										
Anti-GBM			112 (88)		45 (87)		67 (88)	0.79		
Antinuclear antibodies			28 (22)		14 (27)		14 (18)	0.27		
Kidney involvement										
Oligoanuria			80 (63)		35 (67)		45 (59)	0.35		
Peak SCr (µmol/L)			611 [348–1000]		586 [385-1055]		626 [313–975]	0.77		
Peak SCr ≥500 µmol/L			82 (64)		33 (64)		49 (65)	0.91		
Proteinuria > 3.0 (g/24h)			56 (44)		29 (56)		27 (36)	0.02		
Nephrotic syndrome			43 (34)		23 (44)		20 (26)	0.04		
Required dialysis at diagnosis			93 (73)		38 (73)		55 (72)	0.93		
Kidney biopsy findings										
IgG linear GBM deposits			127 (99)		52 (100)		75 (99)	0.41		
IgM linear GBM deposits			7 (6)		4 (8)		3 (4)	0.36		
IgA linear GBM deposits			7 (6)		5 (10)		2 (3)	0.09		
C3 linear GBM deposits			52 (41)		52 (100)		0 (0)	<0.0001		
C1q linear GBM deposits			4 (3)		4 (8)		0 (0)	0.01		
Light chains K/L GBM deposits			21 (16)		13 (25)		8 (11)	0.03		
Light microscopy findings										
Number of normal glomeruli*			0 [0-17]		0 [0–12]		0 [0–21]	0.35		
<10% of normal glomeruli			63 (49)		28 (54)		35 (46)	0.39		
Classification										
-focal			15 (12)		3 (6)		12 (16)	0.08		
-cellular			87 (68)		43 (83)		44 (58)	0.003		
-fibrous			18 (14)		4 (8)		14 (18)	0.09		
-mixed			8 (6)		2 (4)		6 (8)	0.35		
Tubular necrosis and/or interstitial infiltrate			58 (45)		31 (60)		27 (36)	0.007		
Tubular atrophy and/or interstitial fibrosis			25 (20)		7 (14)		18 (24)	0.15		
Lung involvement										
Laboratory variables										
Diffuse alveolar hemorrhage			45 (35)		22 (42)		23 (30)	0.16		
C3 (g/L)			1.23 [1.108-1.38]		1.18 [1.05-1.32]		1.25 [1.10-1.42]	0.06		
C4 (g/L)			0.30 [0.23-0.35]		0.29 [0.22-0.34]		0.30 [0.25-0.35]	0.35		
Serum albumin (g/l)			26.0 [22.0-29.0]		25.2 [22.0-30.7]		26.0 [22.0-28.8]	0.72		
C-reactive protein (mg/l)			102.5 [25.0-178.0]		115.0 [25.0-178.0]		94.5 [25.0-173.5]	0.73		
**Therapeutic management #**			123 (96)		52 (100)		71 (93)			
Intensive treatment			92 (75)		39 (75)		53 (75)	0.96		
Methylprednisolone pulses			123 (100)		52 (100)		71 (100)	0.98		
Plasma exchange			99 (81)		44 (85)		55 (77)	0.32		
Cyclophosphamide			106 (86)		42 (81)		64 (90)	0.14		
Co-trimoxazole prophylaxis			87 (71)		37 (71)		50 (66)	0.70		
**At least one serious infection in the first 12 months of follow-up**			64 (50)		29 (56)		35 (46)	0.28		

Values are expressed as the median [interquartile range] or the number (percentage). The population was limited to the 128 patients with kidney immunostaining data. GBM, glomerular basement membrane antibodies; K/L, kappa/lambda light chains; CST, corticosteroid. * 123 patients with available data, with 52 in the C3+ group and 71 in the C3- group. # The population was limited to the 123 actively treated patients.

### Factor associated with kidney survival

In a univariate analysis, age at diagnosis, peak SCr, oligoanuria and needed dialysis at diagnosis, were associated with one-year kidney survival. Neither the C3 level nor the presence of C3 deposits was associated with kidney survival in the whole population. In a multivariable analysis, only dialysis at diagnosis was independently associated with kidney survival (**Table 5**).

**Table 5:** Factors associated with the kidney outcome in univariate and multivariate Cox regression analyses.

Variable	Univariate analysis		Multivariate analysis	
	HR [95%CI]	*p-value*	HR [95%CI]	*p-value*
Age	1.009 [1.000-1.018]	(p=0.049)	1.005 [0.995-1.014]	(p=0.323)
Gender (female)	1.13 [0.77-1.65]	(p=0.54)		
Charlson Comorbidity Index	1.09 [0.99-1.20]	(p=0.07)		
Oligoanuria	2.71 [1.69-4.35]	(p<0.0001)	0.93 [0.53-1.62]	(p=0.79)
Peak SCr (µmol/L)	1.005 [1.21-2.64]	(p=0.005)	1.000 [0.999-1.001]	(p=0.298)
Proteinuria > 3.0 g/24 h	0.97 [0.66-1.41]	(p=0.86)		
Required dialysis at diagnosis	6.84 [3.31-14.14]	(p<0.0001)	6.19 [2.61; 14.65]	(p<0.0001)
Serum albumin (g/L)	0.99 [0.96-1.02]	(p=0.58)		
C-reactive protein (mg/L)	1.001 [1.000-1.003]	(p=0.14)		
Anti-GBM titre (AU/mL)	1.0001 [0.9994-1,0007]	(p=0.80)		
C3 (g/L)	0.78 [0.41-1.48]	(p=0.45)		
C4 (g/L)	1.21 [0.15-9.94]	(p=0.86)		
Intensive treatment	0.95 [0.62-1.44]	(p=0.80)		
C3 linear GBM deposits	1.13 [0.75-1.71]	(p=0.54)		
Cellular form	1.35 [0.86-2.14]	(p=0.19)		

SCr, serum creatinine; Anti-GBM, antibodies against glomerular basement membrane.

**Figure 4:**
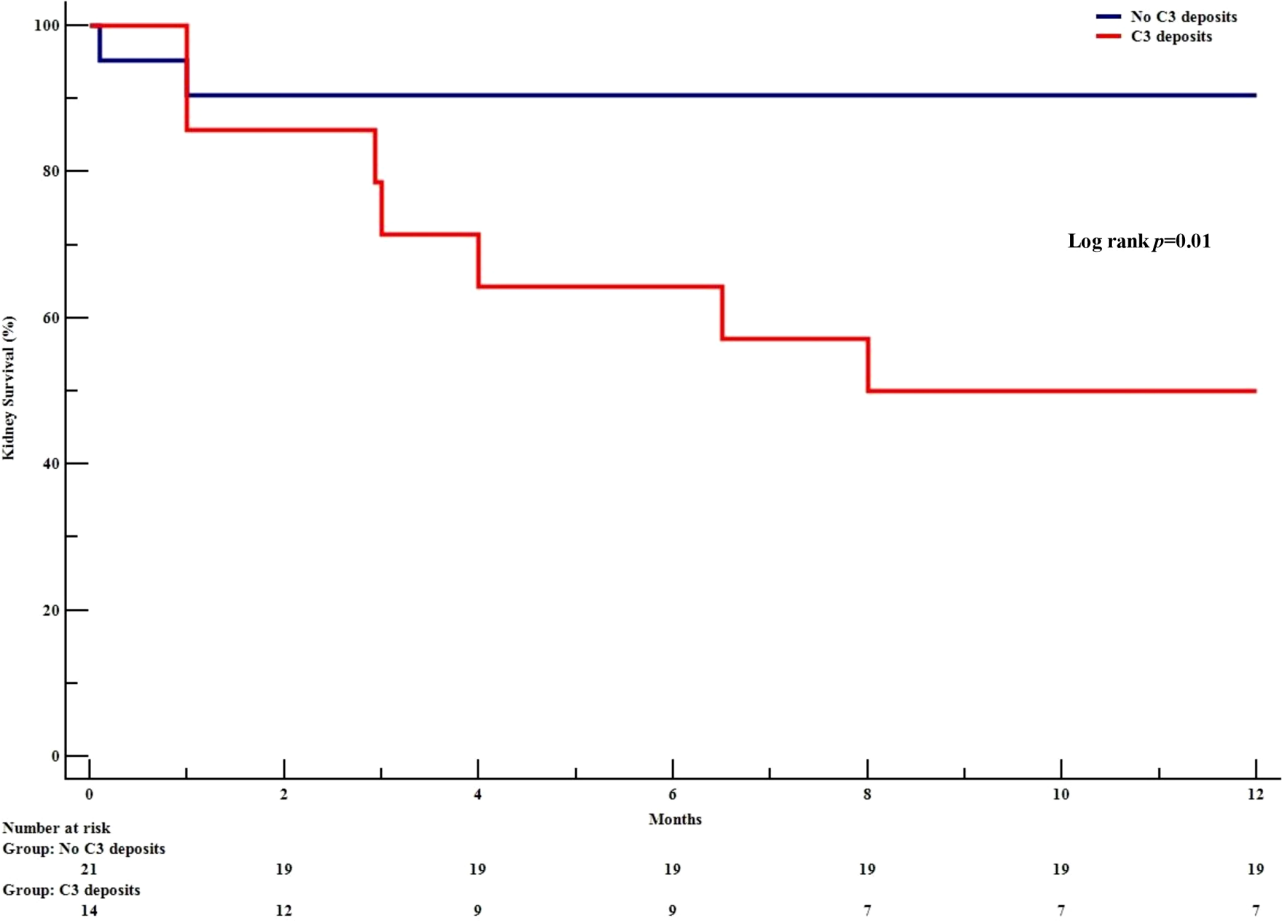
Kaplan Meier kidney survival in initially dialysis-free patients, as a function of the presence or absence of C3 GBM deposits. The Kaplan-Meier survival curve shows the development of end-stage kidney disease in patients with anti-GBM disease and who were not on dialysis at diagnosis, as a function of the presence or absence of C3 GBM deposits.

When considering the subgroup of patients not on dialysis at diagnosis (n=35) (**Supplemental Table 1**) and despite a similar presentation, the kidney survival rate [95%CI] at 12 months was poorer in the C3+ group (50% [25-76]) than in the C3- group (91% [78-100]; p=0.01); the hazard ratio (HR) [95%CI] was 5.98 [1.24-28.89] (p=0.01) (**Figure 4**). After adjusting for SCr, the presence of C3 deposits was still associated with poor kidney survival (HR [95%CI] = 5.71 [1.13-28.85]; p=0.04).

## Discussion

Our present results showed that a low serum C3 level was associated with more severe kidney involvement at diagnosis, as judged from clinical and laboratory data: a greater likelihood of oligoanuria, a peak SCr level ≥500 µmol/L, and nephrotic syndrome. Likewise, the presence of C3 glomerular deposits were also associated with more severe histological damage, as illustrated by the presence of predominant cellular forms and acute tubulo-interstitial lesions. Lastly, the presence of C3 glomerular deposits was associated with worse kidney survival in patients who were not on dialysis at diagnosis.

Several preclinical studies have highlighted the role of complement activation in the pathogenesis of anti-GBM disease (10–12). The selective knockout of C3 and C4 in mouse models of anti-GBM-mediated glomerulonephritis demonstrated the involvement of both the alternative and classical pathways in pathogenesis, with less glomerular neutrophil infiltration and lower proteinuria values, relative to wild-type mice (10, 11). In addition, mice with a deficiency in the classical pathway (C1q−/−) displayed, reduced but persistent C3 deposition and almost the same degree of proteinuria as compared with wild-type mice, indicating an important role of the alternative pathway in complement activation-induced glomerular damage (12). These results also suggest that complement is involved in recruiting inflammatory cells via anaphylatoxins like C3a or C5a. This hypothesis is supported by clinical results, with positive correlations between elevated plasma and/or urine C5a levels on one hand and SCr at presentation and the percentage of crescents in the glomeruli on the other (13). In a pathology-based study of patient samples, Rui et al. showed that even in the absence of C3 deposits, (i) complement activation could be identified by C3d staining, and (ii) the intensities of terminal C5b-9 membrane attack complex (MAC) and factor B glomerular deposits were correlated with the percentage of crescents in the glomeruli - suggesting that MAC is involved in glomerular lesions (14). Furthermore, there is evidence to suggest that C3 can promote T-cell expansion (39), which is known to be involved in the onset of anti-GBM disease (40). The loss of tolerance by CD4+ T-cells in the acute phase of the disease is reestablished by the emergence of regulatory CD25+ T-cells associated with recovery (41, 42). In patients with anti-GBM disease, T-cell infiltration is correlated with kidney damage (43, 44) and poor kidney survival (45). Taken as a whole, these data suggest that C3 contributes to various pathogenic mechanisms in anti-GBM disease and therefore constitutes a potentially valuable therapeutic target.

To the best of our knowledge, the present study is the third to have investigated the prognostic value of the serum C3 level in anti-GBM disease (15, 16). Our results are in line with Zhu et al.’s recent investigation of the association between a low serum C3 at diagnosis and kidney involvement in 94 Chinese patients with anti-GBM disease (16). Low C3 (<0.8 g/l, found in 26 (28%) patients) was associated with a higher proportion of glomerular sclerosis and a poorer kidney involvement, relative to the normal C3 group. Like Zhu et al, we found a higher proportion of the glomerular sclerotic form in the low C3 group. The mechanism underlying this observation is unclear, however, some preclinical data suggest the involvement of complement in the glomerular scarring process through complement-mediated damage (46) or through facilitation of the epithelial-mesenchymal transition (47). Data on time interval between disease onset and diagnosis might help to determine whether this association reflects a longer course of disease in the low C3 group. However, a previous study suggested that the time interval between symptom onset and diagnosis was not predictive of kidney survival (48). Zhu et al. also found an independent association between the C3 level and the kidney prognosis. We failed to show a significant link between the C3 level and the kidney outcome – perhaps because only 10 (6%) patients in our cohort had a C3 level below 0.8 g/l. That is why we chose to dichotomize the population according to the median C3 level in the entire cohort. Although this approach can be criticized (considering that only hypocomplementemia should be considered as a sign of complement activation), it has been used by other researchers in similar settings (24), and C3 has been considered as a continuous variable in statistical analyses. Despite a more severe kidney presentation, patients in the low C3 group were less likely to receive intensive therapy (and notably PLEX); this might reflect a tendency to treat patients with advanced kidney disease less intensively because they are expected to have a poor kidney prognosis. Even though we did not find a significant association between kidney survival and the treatment modality, the latter factor might have biased the outcome in the low C3 group.

In another study, Shen et al. investigated the role of C3 deposits in a cohort of 60 patients presenting with atypical anti-GBM disease (i.e. lacking circulating anti-GBM antibodies). Glomerular C3 deposits were found in 65% of the cohort - compared with 41% in our study (15). Similarly, C3 deposits were associated with greater crescent formation. Moreover, the intensity of kidney C3 staining and a low serum C3 level were independent predictors of poor kidney survival. Despite a more severe kidney presentation in our C3+ group, we failed to find a significant association with kidney survival. This might be related to differences in the study populations’ clinical features. Shen et al. only included patients with atypical anti-GBM disease, which is known to feature less severe kidney disease and a lower prevalence of crescent phenotypes than in classical anti-GBM disease (15, 49, 50). In contrast to Shen et al., we excluded double-positive patients with ANCA because the latter’s course of kidney disease differs from that of single-positive anti-GBM patients (38). The majority of our patients met one or more classical criteria for a poor prognosis (71% were on dialysis, 68% had a cellular form, and 49% had less than 10% of normal glomeruli), which might explain the lack of a difference in kidney survival between the C3+ and C3- groups in the study population as a whole (51, 52). However, when considering only patients not on dialysis at diagnosis, we observed a poorer kidney survival rate at 12 months in the C3+ group - independently of the SCr at diagnosis.

Our study had several limitations. Firstly, the study’s retrospective design might have led to information bias. Kidney pathology assessments were based on each center’s own criteria and were not centrally reviewed. Therefore, we were not able to assess the prognostic value of the intensity of C3 staining, which had been suggested by Shen et al. Likewise, the serum C3 and C4 assay techniques changed during the study period and differed from one center to another; this limited the comparability of the assay results. In our study, the one-year kidney survival rate was low but was in line with recent published cohorts (51, 53). It would be interesting to broaden the cohort to patients with less severe kidney involvement at diagnosis, in order to better evaluate the association between kidney survival on one hand and the serum C3 level and the presence of C3 glomerular deposits on the other. Our study also had a number of strengths. Firstly, our cohort of patients with anti-GBM disease is one of the largest yet described. Secondly, the median follow-up period was relatively long. In line with some other studies, our research highlighted the role of the complement pathway in anti-GBM disease and thus suggested the potential value of anticomplement therapies in this context.

## Conclusion

Our study of patients with anti-GBM disease demonstrated the link between poor kidney health at diagnosis on one hand and a low serum C3 level and the presence of C3 glomerular deposits on the other. Even though we did not find a significant association with kidney or patient survival in the study population as a whole, C3 glomerular deposits were associated with poor outcomes in patients not on dialysis at diagnosis.

## Data availability statement

The raw data supporting the conclusions of this article will be made available by the authors, without undue reservation.

## Ethics statement

The studies involving human participants were reviewed and approved by CPP Nord Ouest II, Amiens, France; reference: TB/LR/2016–91. Written informed consent for participation was not required for this study in accordance with the national legislation and the institutional requirements.

## Author contributions

Conceptualization, data curation, formal analysis, PC and DT-B. Resources, CV, J-MH, MHa, ET, MHe, LJ, VA, MR, AH, J-FS, VV, DG, MT, ME, TL, TQ, ML, AG, M-NP, FV, MD and RM. Supervision, DT-B. Writing—original draft, PC and DT-B. Writing—review & editing, all authors. All authors have read and agreed to the published version of the manuscript.
