# Supplementary material for: Prognostic value of complement serum C3 level and glomerular C3 deposits in anti-glomerular basement membrane disease
Source: Front Immunol. 2023 Jul 5;14:1190394. doi: 10.3389/fimmu.2023.1190394 (PMC10354545; doi:10.3389/fimmu.2023.1190394)
Supplement: Supplementary file 1 [file Table_1.docx]

Supplementary Material

Prognostic value of complement serum C3 level and glomerular C3 deposits in anti-glomerular basement membrane disease

**Pauline Caillard^1,2*^, Cécile Vigneau^3^, Jean-Michel Halimi^4^, Marc Hazzan^5^, Eric Thervet^6^,**

**Morgane Heitz^7^, Laurent Juillard^8^, Vincent Audard^9^, Marion Rabant^10^, Alexandre Hertig^11^, Jean-François Subra^12^, Vincent Vuiblet^13^, Dominique Guerrot^14^, Mathilde Tamain^15^,**

**Marie Essig^16^, Thierry Lobbedez^17^, Thomas Quemeneur^18^, Mathieu Legendre^19^,**

**Alexandre Ganea^20^, Marie-Noëlle Peraldi^21^, François Vrtovsnik^22^, Maïté Daroux^23^, Raïfah Makdassi^1^, Gabriel Choukroun^1,2^, Dimitri Titeca-Beauport^1,2^**

*** Correspondence:**

Pauline Caillard
caillard.pauline@chu-amiens.fr

# Supplementary Figures and Tables

| **Supplemental Table 1. Clinical and histological characteristics at diagnosis, as function of the presence or absence of C3 kidney deposits in the subgroup of patients not on dialysis at diagnosis** | | | | | |
| --- | --- | --- | --- | --- | --- |
|  | **Total**  (n=35) | **C3+**  (n=14) | **C3-**  (n=21) | ***p*-value** | |
| Age | 33 [23–52] | 26 [22–45] | 43 [26–55] | 0.11 | |
| **Antibodies** | | | | | |
| Anti-GBM | 29 (83) | 13 (93) | 16 (76) | 0.21 | |
| **Kidney involvement** | | | | | |
| Oligoanuria | 1 (3) | 1 (7) | 0 (0) | 0.22 | |
| Peak SCr (µmol/L) | 242 [154–354] | 282 [238-450] | 209 [139–345] | 0.18 | |
| Peak SCr ≥500 µmol/L | 2 (6) | 1 (7) | 1 (7) | 0.77 | |
| Proteinuria > 3.0 (g/24h) | 18 (51) | 9 (64) | 9 (43) | 0.22 | |
| **Kidney biopsy findings** | | | | | |
| Classification |  |  |  |  | |
| -focal | 11 (31) | 2 (14) | 9 (43) | 0.08 | |
| -cellular | 19 (54) | 10 (72) | 9 (43) | 0.10 | |
| -fibrous | 0 (0) | 0 (0) | 0 (0) |  | |
| -mixed | 5 (15) | 2 (14) | 3 (14) | 1.00 | |
| **Lung involvement** |  |  |  |  | |
| Diffuse alveolar hemorrhage | 12 (34) | 7 (50) | 5 (24) | 0.12 | |
| **Laboratory variables** | | | | | |
| C3 (g/L) | 1.30 [1.11-1.51] | 1.27 [1.07-1.39] | 1.37 [1.12-1.52] | | 0.23 |
| C4 (g/L) | 0.30 [0.25-0.35] | 0.28 [0.20-0.31] | 0.30 [0.26-0.36] | | 0.24 |
| Serum albumin (g/l) | 25.0 [22.0-28.6] | 26.5 [22.0-34.0] | 24.0 [22.8-28.0] | | 0.29 |
| C-reactive protein (mg/l) | 51.0 [15.5-130.0] | 56.5 [20.0-140.0] | 28.0 [4.9-113.3] | | 0.42 |
| **Therapeutic management** | 35 (35) | 14 (100) | 21 (100) | |  |
| Intensive treatment | 26 (74) | 10 (71) | 16 (76) | | 0.76 |
| Plasma exchange | 27 (77) | 11 (79) | 16 (76) | | 0.87 |
| Values are expressed as the median [interquartile range] or the number (percentage). Anti-GBM: antibodies against glomerular basement membrane. | | | | | |
